# Preoperative and postoperative anemia in major elective surgery: insights from a retrospective cohort in a Brazilian University Hospital

**DOI:** 10.1016/j.bjane.2025.844715

**Published:** 2025-11-22

**Authors:** Lorena dos Santos Goiabeira, Sara Silva Meireles, Allan Santos Silva Leocádio, Heitor J.S. Medeiros, Fernanda Cunha Soares, Aline Macedo Pinheiro, Wallace Andrino da Silva

**Affiliations:** a Universidade Federal do Rio Grande do Norte, Natal, RN, Brazil; b Massachusetts General Hospital, Department of Anesthesia, Critical Care and Pain Medicine, Boston, USA; c Karolinska Institutet, Department of Dental Medicine, Division of Orthodontics and Pediatric Dentistry, Stockholm, Sweden; d Hospital Universitário Onofre Lopes, Departamento de Cirurgia, Disciplina de Anestesiologia, Natal, RN, Brazil

**Keywords:** Anemia, Blood transfusion, Intraoperative period

## Abstract

**Background:**

Anemia is a common and critical condition in the perioperative management of patients undergoing major elective surgeries, posing significant risks to postoperative recovery. This study aimed to evaluate the prevalence of preoperative and postoperative anemia in surgical patients from a university hospital in northeastern Brazil.

**Methods:**

This retrospective study included 508 patients aged 18 years or older who underwent major elective surgeries between October 2021 and October 2022. Anemia was defined according to World Health Organization criteria (hemoglobin < 13 g.dL^-1^ for men and < 12 g.dL^-1^ for women). Data were extracted from medical records and included preoperative and postoperative hemoglobin levels, surgical types, and transfusion requirements.

**Results:**

Preoperative anemia was observed in 59.6% of 508 patients analyzed, with a mean Hb level of 11.66 (±/ 2.75) g.dL^-1^ and 11.13 (± 2.08) g.dL^-1^ for women and men, respectively. In the postoperative period, the anemia rate increased to 94.6%, with a mean Hb level of 9.36 (± 1.55) g.dL^-1^ and 9.49 (± 1.36) g.dL^-1^ for women and men, respectively. The transfusion rate was 27% in the total sample. Patients with preoperative anemia were 4.6 times more likely to require intraoperative transfusion compared to non-anemic patients (OR = 4.58; 95% CI: 2.78–7.52; p < 0.001). Higher preoperative hemoglobin levels were identified as protective against transfusion (OR = 0.65; 95% CI: 0.59–0.72; p < 0.001).

**Conclusions:**

Preoperative anemia is a highly prevalent and modifiable risk factor associated with increased transfusion requirements and adverse perioperative outcomes. The study highlights the importance of implementing patient blood management protocols in surgical practice.

## Introduction

Anemia is a critical condition in the perioperative management of patients undergoing elective surgeries. Defined by the World Health Organization (WHO) as Hemoglobin (Hb) levels < 13 g.dL^-1^ in men and < 12 g.dL^-1^ in women, preoperative anemia is a prevalent comorbidity affecting up to 40% of surgical candidates and poses significant risks to postoperative recovery.^1^ The etiology is often multifactorial, including chronic disease, iron deficiency, and inflammatory states. Notably, preoperative anemia serves as an independent predictor of adverse outcomes, including increased mortality, prolonged hospitalization, and higher rates of postoperative complication.^2^^,^^3^

Postoperative anemia is even more prevalent than its preoperative counterpart, with rates reaching 80%–90% after major surgeries. The condition arises from perioperative blood loss, hemodilution, and inflammation-induced suppression of erythropoiesis. Elevated levels of hepcidin, a regulatory protein of iron metabolism, further impair iron availability, delaying Hb recovery.^2^^,^^3^

The recognition of anemia as a modifiable risk factor has prompted the adoption of Patient Blood Management protocols, which encompass three pillars: 1) Detection and treatment of preoperative anemia, 2) Minimization of intraoperative blood loss, and 3) Optimization of patient-specific anemia tolerance.^4^

This study aimed to conduct an epidemiological assessment of preoperative and postoperative anemia cases in elective major surgeries performed at a university hospital in northeastern Brazil.

## Methods

This was a retrospective study conducted between October 2021 and October 2022. The project was submitted to and approved by the Research Ethics Committee under protocol number CAAE 67056522.7.0000.5292.

### Study population and data collection

All patients aged 18 years or older who underwent elective surgery and had blood component reservations between October 2021 and October 2022 were included in the study. According to international and institutional protocol, blood components are reserved for major surgeries and/or procedures with an estimated blood loss of 500 mL or greater.^5^

Data were collected from both electronic and physical medical records archived at the University Hospital Onofre Lopes. The following variables were extracted: sex, age, type of surgical procedure, and preoperative and postoperative Hb levels (g.dL^-1^). Preoperative Hb levels were defined if collected 24–48 hours prior to surgery, while postoperative levels were defined if collected on the first postoperative day. The occurrence of intraoperative red blood cell concentrate transfusion was confirmed through data from the hospital's Blood Bank.

Anemia was defined according to the WHO criteria: Hb levels < 13 g.dL^-1^ in males and < 12 g.dL^-1^ in females.^6^ There were no pregnant women enrolled in this study.

The surgical procedures analyzed included cardiovascular, oncological, neurological, and urological surgeries, as well as a subgroup categorized as “other”. This subgroup encompassed gastrointestinal, orthopedic, thoracic, and other surgical procedures.

### Statistical analysis

The analyses were conducted using STATA software, version 14 (StataCorp LP, College Station, USA) for Windows. Measures of central tendency and dispersion were presented as means and standard deviations. Frequency distributions were employed for variables measured on a nominal scale.

Data distribution and completeness were assessed prior to analysis. Missing data were handled using listwise deletion (complete case analysis), wherein entire records were excluded if any variable had missing values.

Comparisons between means were performed using Student’s *t*-test, while associations between qualitative variables were analyzed using the Chi-Square test. A p-value < 0.05 was considered statistically significant for all two-tailed tests.

Multivariate analysis was performed using “need for blood transfusion” as the primary outcome variable. Covariates were selected based on two criteria: a) Variables with a p-value < 0.05 in the bivariate analysis; b) Variables reported in the literature as key covariates (e.g., age and sex). Binary logistic regression was used to evaluate the association between preoperative Hb levels or anemia and the occurrence of transfusion. The logistic regression model was adjusted for sex and age. Odds Ratios (ORs) with 95% Confidence Intervals (95% CIs) were calculated, and statistical significance was determined at a p-value < 0.05.

## Results

**Figure 1:**
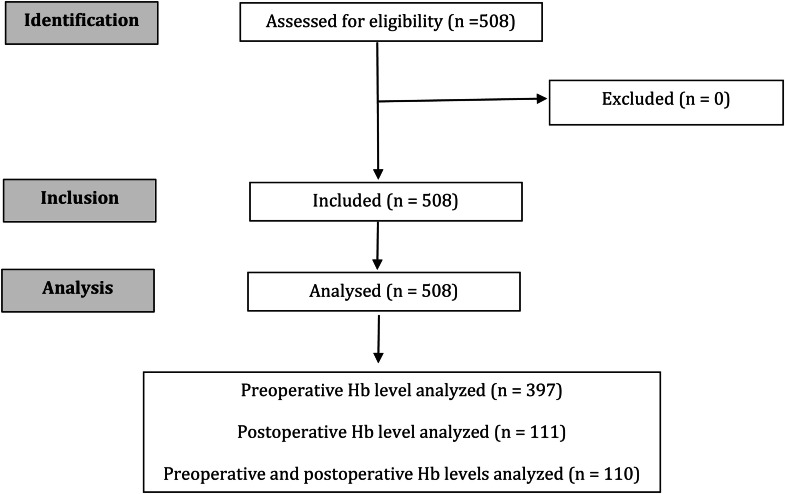
STROBE diagram showing the flow of patients in the study.

A total of 508 patients undergoing major elective surgeries were analyzed. Among them, 397 had only preoperative hemoglobin data, 111 had only postoperative data, and 110 had both preoperative and postoperative hemoglobin measurements, as shown in Figure 1.

Of the total sample analyzed, the mean age was 57.1 (± 14.4) years, of whom 51% were male. The overall rate of intraoperative transfusion was 27%. Preoperative anemia was more prevalent in urological surgeries (73%). In the preoperative evaluation, anemia was observed in 59.6% of 508 patients analyzed, with a mean Hb level of 11.66 (±/ 2.75) g.dL^-1^ and 11.13 (± 2.08) g.dL^-1^ for women and men, respectively. In the postoperative period, among 110 patients evaluated, the anemia rate increased to 94.6%, with a mean Hb level of 9.36 (± 1.55) g.dL^-1^ and 9.49 (± 1.36) g.dL^-1^ for women and men, respectively.

When analyzing preoperative anemia, patients with anemia were older (58.0 ± 14.0 years). Among those with preoperative anemia, the highest prevalence was observed in patients who underwent urological surgery (73%), and most of these patients required blood transfusions (83%). There was no significant difference between male and female patients. Regarding postoperative anemia, no association was found with any of the studied variables (Table 1).

**Table 1:** Characteristics and prevalence of the patients with preoperative and postoperative anemia.

Variables	Total (n = 508)	Preoperative anemia			Postoperative anemia		
		No (n = 205)	Yes (n = 302)	p-value	No (n = 6)	Yes (n = 105)	p-value
Sex, n (%)							
Female	250 (49)	100 (40)	150 (60)	0.844	3 (6)	50 (94)	0.910
Male	258 (51)	105 (41)	152 (59)		3 (5)	55 (95)	
Age (years), mean ± SD	57.1 ± 14.4	54.4 ± 14.8	58.0 ± 14.0	**< 0.001**	61.8 ± 12.3	58.0 ± 12.8	0.483
Surgery, n (%)							
Cardiovascular	131 (26)	60 (46)	71 (54)	**< 0.001**	3 (10)	28 (90)	0.210
Neurological	24 (5)	14 (61)	9 (39)		0 (0)	5 (100)	
Oncological	134 (26)	67 (50)	67 (50)		0 (0)	14 (100)	
Urological	136 (27)	37 (27)	99 (73)		0 (0)	34 (100)	
Others	83 (16)	27 (33)	56 (67)		3 (11)	24 (89)	
Intraoperative transfusion^a^, n (%)							
No	371 (73)	181 (49)	190 (51)	**< 0.001**	0 (0)	0 (0)	‒
Yes	135 (27)	23 (17)	111 (83)		6 (5)	105 (95)	

a One or more red blood cell unit.

**Figure 2:**
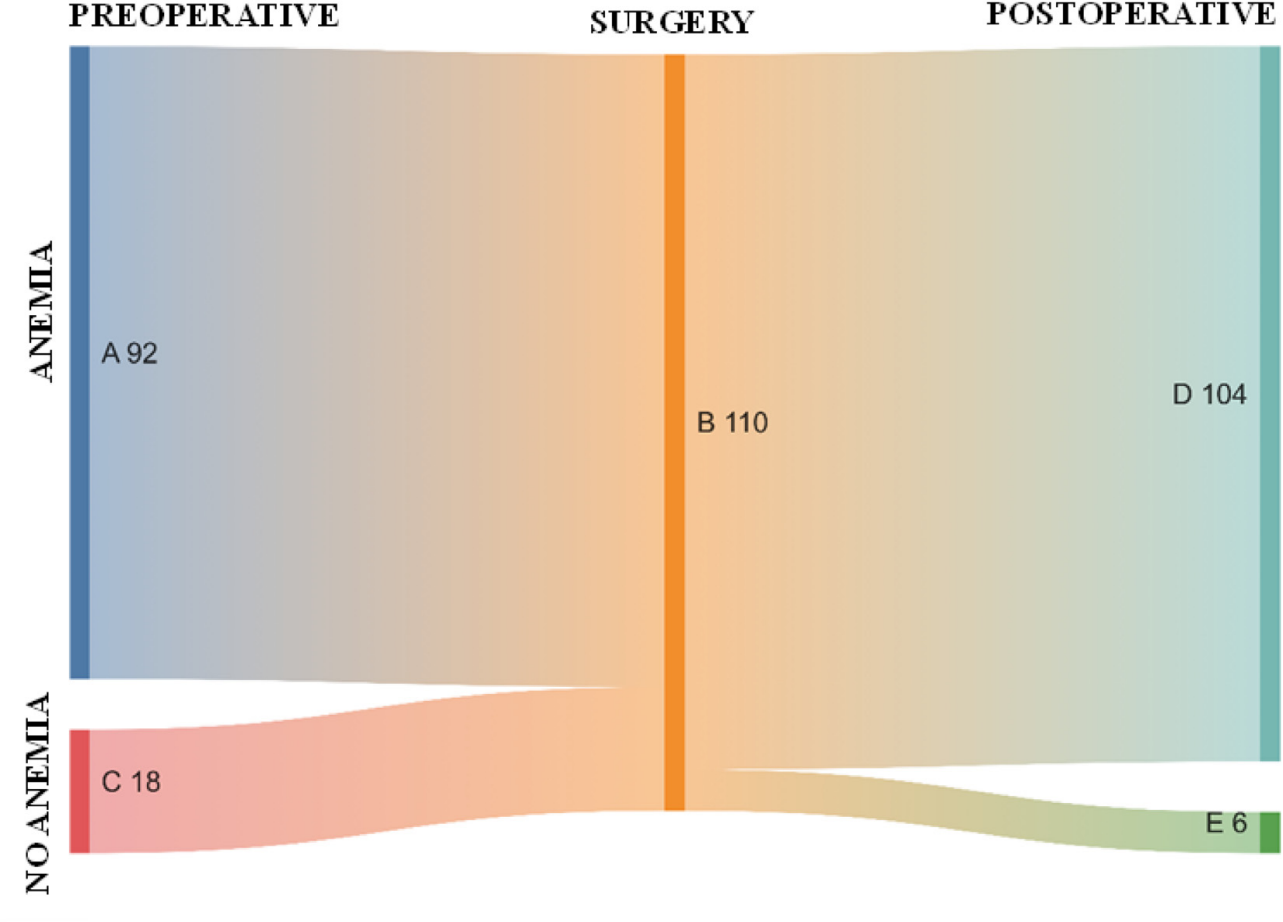
Sankey diagram illustrating the perioperative development of anemia status in the 110 patients analyzed in the study. (A) 92 patients with preoperative anemia; (B) 110 patients who underwent surgery; (C) 18 patients without preoperative anemia; (D) 104 patients with postoperative anemia (90 from preoperative anemia group and 14 from preoperative no anemia group); (E) 6 patients without postoperative anemia (4 from preoperative no anemia group and 2 from preoperative anemia group).

Among the 110 patients with Hb levels monitored both preoperatively and postoperatively, 92 (83.6%) had preoperative anemia. Of these, 97.8% (90 patients) remained anemic postoperatively, while only 2.2% (2 patients) no longer exhibited anemia postoperatively. Among the 18 (16.4%) patients without preoperative anemia, 22.2% (4 patients) remained non-anemic postoperatively, whereas 77.8% (14 patients) developed anemia in the postoperative period (Fig. 2).

Multivariate analysis revealed that patients with preoperative anemia were approximately 4.6 times (95% CI: 2.78–7.52) more likely to require red blood cell transfusions compared to those without preoperative anemia. In a second regression model, higher preoperative Hb levels were identified as a protective factor against the need for blood transfusion (OR = 0.65; 95% CI: 0.59–0.72) (Table 2).

**Table 2:** Adjusted logistic regression between preoperative hemoglobin and anemia with intraoperative red blood cell transfusion.

	Model I^b^	p-value	Model II^b^	p-value
	OR (95% CI)		OR (95% CI)	
Anemia^a^				
No	1	**< 0.001**	‒	-
Yes	4.58 (2.78:7.52)		‒	
**Preoperative Hb (g.dL-1)**	‒	‒	0.65 (0.59:0.72)	**< 0.001**

a Hb < 13 g.dL^-1^ in males and < 12 g.dL^-1^ in females.

b Adjusted for sex and age.

## Discussion

Our study revealed that over 50% of patients presented with preoperative anemia, and more than 90% experienced anemia in the postoperative period following major elective surgery. These findings align with global literature, where the average prevalence of preoperative anemia is approximately 40%, and postoperative anemia is reported in around 90% of cases.^1^^,^^2^^,^^7^, ^8^, ^9^

A retrospective cohort study conducted in a public hospital in Brazil examined the prevalence of preoperative anemia and its impact on postoperative outcomes among 15,166 surgical patients. The study found that preoperative anemia was prevalent (42.1% of the population studied) and was associated with increased postoperative complications, including higher morbidity and mortality rates. These results underscore the importance of early detection and management of anemia in surgical patients to improve postoperative outcomes.^10^

Another Brazilian study, conducted across 16 blood centers, analyzed data from over 20,000 surgical patients to assess the burden of perioperative anemia and its implications for surgical care. This study reported a prevalence of preoperative anemia of 60.9% within the cohort. Key findings included gender disparities (66.9% of women had anemia), age-related trends (68.2% of patients over 65 years exhibited the highest anemia prevalence), and regional variability (64% in the Northeast, 66.3% in the South, and 63.9% in the Southeast). Notably, there was no significant correlation between anemia prevalence and the Human Development Index of the regions, suggesting that anemia affects diverse socioeconomic strata.^11^

Preoperative anemia is an independent predictor of adverse outcomes, including increased mortality, prolonged hospitalization, and higher rates of postoperative complications.^2^^,^^3^ A meta-analysis of over 900,000 patients demonstrated that preoperative anemia is associated with nearly a threefold increase in 30-day mortality (Odds Ratio [OR = 2.90]), as well as elevated risks of acute kidney injury (OR = 3.75) and postoperative infections (OR = 1.93).^3^ Additionally, patients with anemia are more likely to require perioperative blood transfusions, which are linked to poorer outcomes. These findings highlight the critical need for routine preoperative anemia screening and management as part of Patient Blood Management (PBM) strategies.^2^^,^^3^ However, significant gaps remain, as nearly 25% of patients undergoing elective surgeries are not evaluated for anemia.^12^ Standardized protocols for anemia management are essential to improve surgical outcomes.

Postoperative anemia is a common complication after major surgery due to the inflammatory response triggered by surgical trauma.^2^ The consequences of postoperative anemia are significant, with studies linking it to increased morbidity, delayed functional recovery, and higher rehospitalization rates.^1^ In cardiac and orthopedic surgeries, postoperative anemia has been associated with myocardial infarction, prolonged rehabilitation, and higher mortality rates.^12^ Management strategies include judicious use of blood transfusions, which, while effective in rapidly increasing hemoglobin levels, carry risks such as immunosuppression and infection.^1^

Oral iron therapy has traditionally been the first-line treatment for perioperative anemia, but its effectiveness is limited by poor absorption in inflammatory states, gastrointestinal side effects, and delayed onset of action. Consequently, intravenous iron has emerged as a more effective alternative, allowing rapid replenishment of iron stores and hemoglobin synthesis. Modern intravenous iron preparations are associated with low risks of adverse reactions and are considered safe in the perioperative setting.^1^^,^^13^

The PREVENTT trial demonstrated that preoperative intravenous iron increased perioperative hemoglobin levels and reduced hospital readmissions, although it did not significantly reduce perioperative blood transfusion rates or mortality.^14^

Considering these findings, the implementation of structured PBM programs becomes particularly relevant in our local context. The high prevalence of preoperative anemia, coupled with the very high rates of postoperative anemia and the observed association between preoperative anemia and intraoperative transfusion, underscores the need for systematic strategies to optimize patients’ hematologic status before surgery. Practical measures include routine preoperative screening for anemia, timely initiation of iron supplementation (preferably intravenous in patients with limited surgical timelines or inflammatory states), and standardized perioperative transfusion protocols tailored to minimize unnecessary exposure to allogeneic blood products. Integrating these measures into surgical pathways could not only reduce transfusion requirements but also improve functional recovery, shorten hospital stays, and mitigate postoperative complications. In resource-constrained settings such as ours, strengthening PBM initiatives offers a cost-effective and evidence-based approach to enhance surgical safety and outcomes.^15^

Recommendations from the International Consensus Conference on Anemia Management in Surgical Patients emphasize the importance of preoperative anemia screening, the evaluation of postoperative anemia, and the strategic use of intravenous iron during the perioperative period to optimize patient outcomes.^16^

This study is subject to several potential sources of bias inherent to its retrospective design. Selection bias may have occurred given that only patients with reserved blood components for major elective surgeries were included, potentially excluding cases of comparable surgical magnitude without preoperative reservations. Information bias is also a concern, particularly due to reliance on medical records, which may contain inconsistencies or omissions in hemoglobin measurements and transfusion data. Additionally, missing data ‒ especially regarding postoperative hemoglobin levels, which were unavailable for a substantial portion of the cohort ‒ may have introduced bias and limited the ability to comprehensively assess perioperative hemoglobin dynamics. Furthermore, our logistic regression model was adjusted only for age and sex because data on other potential confounders, such as comorbidities and surgical complexity, were not consistently available due to the retrospective design of the study. Furthermore, the sample size limited the inclusion of additional variables without risking model overfitting.

## Conclusions

This study revealed a high prevalence of both preoperative and postoperative anemia among patients undergoing major elective surgeries in northeastern Brazil. The association between preoperative anemia and increased intraoperative transfusion rates underscores anemia as a modifiable and clinically relevant risk factor. These findings reinforce the urgent need for systematic preoperative anemia screening and proactive treatment strategies, particularly intravenous iron therapy, as integral components of patient blood management PBM programs. Implementing these measures can optimize preoperative hemoglobin levels, reduce transfusion requirements, and ultimately improve surgical outcomes. Future studies should prioritize prospective designs and long-term follow-up to assess the sustained impact of anemia correction within PBM frameworks.

## Data availability statement

The datasets generated and/or analyzed during the current study are available from the corresponding author upon reasonable request.

## AI assistance disclosure

No Artificial Intelligence (AI) tools were used in the preparation, writing, editing, or analysis of this manuscript. All content was produced entirely by the authors, who take full responsibility for the accuracy and integrity of the work.

## Ethics approval and consent to participate

Ethical approval was granted by the Onofre Lopes University Hospital Research Ethics Committee, under the Ethical Appreciation Presentation Certificate number CAAE 67056522.7.0000.5292, approved on April 7, 2023.

## Declaration of competing interest

The authors declare no conflicts of interest.
